# Slower immune system aging in women versus men in the Japanese population

**DOI:** 10.1186/1742-4933-10-19

**Published:** 2013-05-15

**Authors:** Katsuiku Hirokawa, Masanori Utsuyama, Yoshio Hayashi, Masanobu Kitagawa, Takashi Makinodan, Tamas Fulop

**Affiliations:** 1 Institute for Health and Life Sciences, Tokyo Medical & Dental University Open Laboratory, Medical Research Institute Surugadai Bldg, 2-3-10, Surugadai, Kanda, Chiyoda-ku, Tokyo 101-0062, Japan; 2 Department of Comprehensive Pathology, Tokyo Medical & Dental University, Tokto, Japan; 3 Nakanosogo Hospital, Tokto, Japan; 4 UCLA, Los Angeles, USA; 5 Research Center on Aging, University of Sherbrooke, Sherbrooke, Canada

**Keywords:** Ageing, Immune system, Gender difference, T cells, T cell immune score, T cell proliferation index, CD8^+^CD28^+^ T cells

## Abstract

**Background:**

Gender-related differences in humans are commonly observed in behaviour, physical activity, disease, and lifespan. However, the notion that age-related changes in the immune system differ between men and women remains controversial. To elucidate the relationship between immunological changes and lifespan, peripheral blood mononuclear cells from healthy Japanese subjects (age range: 20–90 years; N = 356) were analysed by using three-colour flow cytometry. The proliferative activities and cytokine-producing capacities of T cells in response to anti-CD3 monoclonal antibody stimulation were also assessed.

**Results:**

An age-related decline in the number of T cells, certain subpopulations of T cells (including CD8^+^ T cells, CD4^+^CDRA^+^ T cells, and CD8^+^CD28^+^ T cells), and B cells, and in the proliferative capacity of T cells was noted. The rate of decline in these immunological parameters, except for the number of CD8^+^ T cells, was greater in men than in women (p < 0.05). We observed an age-related increase or increasing trend in the number of CD4^+^ T cells, CD4^+^CDRO^+^ T cells, and natural killer (CD56^+^CD16^+^) cells, as well as in the CD4^+^ T cell/CD8^+^ T cell ratio. The rate of increase of these immunological parameters was greater in women than in men (p < 0.05). T cell proliferation index (TCPI) was calculated from the T cell proliferative activity and the number of T cells; it showed an age-related decline that was greater in men than in women (p < 0.05). T cell immune score, which was calculated using 5 T cell parameters, also showed an age-related decline that was greater in men than in women (p < 0.05). Moreover, a trend of age-related decreases was observed in IFNγ, IL-2, IL-6, and IL-10 production, when lymphocytes were cultured with anti-CD3 monoclonal antibody stimulation. The rate of decline in IL-6 and IL-10 production was greater in men than in women (p < 0.05).

**Conclusion:**

Age-related changes in various immunological parameters differ between men and women. Our findings indicate that the slower rate of decline in these immunological parameters in women than that in men is consistent with the fact that women live longer than do men.

## Background

Immunological functions are known to decline with age in animal models and humans [1-3]. An understanding of immunological functions at an individual level is clinically important because immunological decline is accompanied by various ailments such as infections, cancer, vascular diseases, and possibly autoimmune phenomena in the aged.

The concept of immunosenescence reflects changes in both cellular and humoral immune responses. Accumulating evidence obtained mainly from animal models has shown that age-related immunological decline occurs primarily in T cell-dependent immune functions, and it is mainly caused by thymic involution that begins in the early phase of life [3]. As a result, T cells are central to the immune response, and their function is significantly altered with increasing age. In humans, data on immunological functions are mainly obtained from blood serum and blood cells. Serum contains immunoglobulin, complements, and cytokines. The levels of serum IgG and IgA generally tend to increase slightly with age [4], while the level of serum complements does not change remarkably with age. The level of cytokines in healthy old people is generally reduced. In contrast, the level of white blood cells (WBCs) remarkably changes with disease and aging. WBCs are constituted of lymphocytes, monocytes, and neutrophils. There are various subpopulations of lymphocytes with different functions. Data on age-related changes in human lymphocytes and their functions are limited.

Besides aging, the fact that gender influences the immune system has long been recognised. In postmenopausal women, changes in the immune system have been attributed to oestrogen deprivation. Women are at higher risk for developing autoimmune diseases, which suggests that these diseases are somehow mediated by sex steroids, with oestrogens as enhancers, at least with humoral immunity. There is an increase in proinflammatory serum markers, an increasing response of the body’s cells to cytokines, a decrease in CD4^+^ T and B lymphocytes, and in the cytotoxic activity of natural killer (NK) cells [5]. One cellular process related to lifespan, which differs according to sex, is the rate at which the protective telomere chromosome caps are lost. In humans, men have shorter lifespans and greater telomere shortening than do women. This has led to speculation in the medical literature that sex-specific telomere shortening is one cause of sex-specific mortality [6].

Therefore, the purpose of this study is to obtain data on age-related changes and gender differences in the number and function of peripheral blood lymphocytes and their subpopulations obtained from 162 healthy male and 194 healthy female volunteers, ranging in age from 20 to 90 years. The results show that the rate of age-related immunological decline is less in women than in men, and this could be a contributory factor to women living longer than men in Japan.

## Results

1) Age-related changes in RBCs and WBCs (Table 1)

A significant age-related decrease in the number of RBCs was observed in men (p < 0.001) but not in women (Table 1), although absolute levels of RBCs in subjects over 60 years was significantly higher in men than in women (Table 2). The age-related difference in the number of RBCs between men and women was statistically significant (p < 0.001).

The number of neutrophils showed a decreasing trend with age in both men (slope: -71.31, p = 0.218) and women (slope: -70.39, p = 0.009), but no significant difference was observed (Table 1).

The number of lymphocytes showed a decreasing trend with age in men (slope: -43.30, p = 0.059) and an increasing trend with age in women (slope: 33.05, p = 0.996), and the difference between genders was statistically significant (p = 0.016, Table 1). Therefore, the number of lymphocytes was higher in young men than in young women (data, not shown), but the absolute number of T cells in people over 60 years was almost the same between men and women (Table 2).

2) Immunological parameters showing age-related decline (Table 3)

(a) T cells.

**Figure 1:**
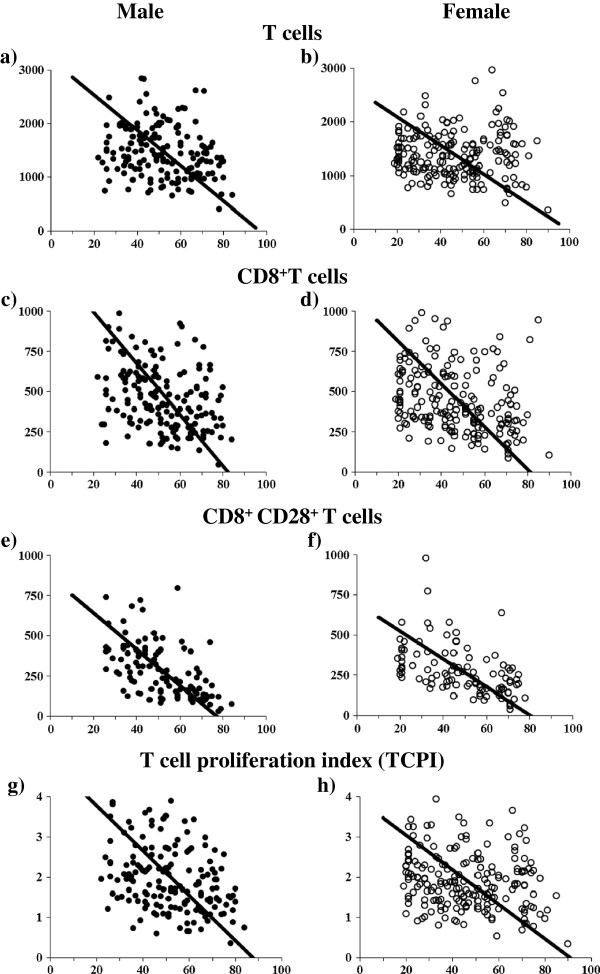
**Age-related change in the number of T cells, CD8**^**+ **^**T cells, CD8**^**+**^**CD28**^**+ **^**T cells and TCPI in men and women.** The slopes of T cells are -33.00 (**a**: men) versus -26.57 (**b**: women). The slopes of CD8^+^ T cells are -15.94 (**c**: men) versus -13.24 (**d**: women). The slopes of CD8^+^CD28^+^ T cells are -11.21 (**e**: men) versus -8.66 (**f**: women). The slopes of TCPI are -0.056 (**g**: men) versus -0.043 (**h**: women). Gender difference is observed in T cells (p = 0.049), CD8^+^CD28^+^ T cells (p = 0.04) and TCPI (p = 0.01).

The number of T cells (CD3^+^) decreased significantly with age in men (slope: -33.00, p = 0.018), and had a decreasing trend with age in women (slope: -26.57, p = 0.212). The rate of decline with age was greater in men than in women (p = 0.049, Figure 1a and b, Table 3).

(b) CD8^+^T cells.

A subpopulation of CD8^+^ T cells showed an age-related decrease in both men (slope: -15.49, p < 0.001) and women (slope: -13.24, p < 0.001), while the magnitude of the decrease between them was not statistically significant (Figure 1c and d, Table 3).

(c) CD8^+^CD28^+^ T cells.

A subpopulation of CD8^+^CD28^+^ T cells showed an age-related decrease in both men (slope: -11.21, p < 0.001) and women (slope: -8.664, p < 0.001, Fig. 1e and f), and the rate of this decline was greater in men than in women (p < 0.04, Table 3).

(d) Naïve T cells.

The number of naïve T cells (CD4^+^CD45RA^+^ T cells) showed a decreasing trend with age in both men (slope: -15.39, p = 0.261) and women (slope: -10.88, p = 0.447, Table 3), which was greater in men than women; the difference between them was statistically significant (p = 0.001).

(e) T cell proliferative activity.

A proliferative response of T cells to anti-CD3 monoclonal antibody stimulation was measured by the MTS method and was expressed as OD_490_ (MTS-OD_490_). The results showed an age-related decrease in both men (slope: -0.019, p < 0.001) and women (slope: -0.016, p = 0.002, Table 3), but no gender difference was observed.

(f) T cell proliferation index (TCPI).

TCPI showed an age-related decrease in both men (slope: -0.056, p < 0.001) and women (slope: -0.043, p = 0.008, Fig. 1g and h). The decrease was greater in men (slope: -0.056) than in women (p = 0.01, Table 3).

(g) T cell immune score.

The T cell immune score was calculated by using 5 parameters as described in the Methods. The T cell immune score showed an age-related decrease in both men (slope: -0.137, p = 0.001) and women (slope: -0.107, p < 0.001, Table 3). A greater decrease was observed in men than women (p = 0.006).

(h) B cells.

The number of B cells (i.e. CD20^+^ lymphocytes) showed a decrease with age in men (slope: -11.04, p = 0.034) and showed a decreasing trend with age in women (slope: -4.307, p = 0.152); the magnitude of the decrease between men and women was statistically significant (p = 0.001, Table 3).

3) Immunological parameters showing age-related increases (Table 4)

(a) CD4^+^ T cells.

A subpopulation of CD4^+^ T cells was maintained at steady state in men (slope:23.28, p=0.9897) and showed an increasing trend with age in women (slope: 16.92, p=0l075). The slope was greater in men than in women, and the difference between them was statistically significant (p = 0.005, Table 4).

(a) The ratio of CD4^+^ T cells to CD8^+^ T cells (CD4/CD8 ratio).

The CD4/CD8 ratio increased with age in both men (slope: 0.087, p < 0.001) and women (slope: 0.064, p < 0.001), and this increase was significantly greater in men than women (p = 0.001, Fig. 2g and 2h, Table 4).

(a) Memory T cells.

The number of memory T cells (CD4^+^CD45RO^+^) showed an increasing trend with age in men (slope: 12.35, p = 0.153), and a significant age-related increase in women (slope: 10.11, p < 0.001); the magnitude of increase was significantly greater in men than in women (p < 0.001, Table 4).

(a) CD4^+^CD25^+^ T cells.

A subpopulation of CD4^+^CD25^+^ T cells showed an increasing trend with age in both men (slope: 1.83, p = 0.379) and women (slope: 1.602, p = 0.233), but no gender difference was observed (Table 4).

(a) NK cells.

The number of NK cells (CD56^+^CD16^+^) showed an age-related increase in women (slope: 11.94, p < 0.001) and an increasing trend with age in men (slope: 17.41, p = 0.194). The magnitude of the increase was significantly greater in men than in women (p < 0.001, Table 4).

4) Cytokine production (Table 5)

The production of IFNγ, IL-2, IL-6, and IL-10 showed a decreasing trend with age, although the results were not statistically significant, with the exception of IL-6 in men (Table 5). However, SAM analysis revealed that there was a significant difference in IL-6 and IL-10 between men and women. The slope of IL-6 was greater in women (-488.0) than in men (-293.0), and the slope of IL-10 was greater in men (-51.4) than in women (-28.1).

**Table 1:** Age-related change of RBC and WBC in male and female subjects

**Hematological parameters**	**Men (N = 162)**				**Women (N = 194)**				**Gender difference**
	**Slope**	**Intercept**	**R**	**p-value**	**Slope**	**Intercept**	**R**	**P-value**	**p-value**
RBC^※^	−0.033	6.37	0.517	0.001	−0.021	5.238	0.105	0.138	0.001
WBC^※^	−0.099	11.27	0.138	0.082	−0.091	9.980	0.155	0.033	NS
Lymphocytes^※^	−43.30	4455	0.148	0.059	33.05	452	<0.001	0.996	0.016
Neutrophils^※^	−71.31	7087	0.095	0.218	−73.39	6202	0.187	0.009	NS

※:Number / mm3. R: Correlation coefficient. NS: not significant.

**Table 2:** Gender difference in people over 60 years old

**Immunological parameters**	**Men**	**Women**
	N = 54	N = 48
T cells (number/mm^3^)	1365 ± 53	1395 ± 73
CD4^+^ T cells (number/mm^3^)	910 ± 59	961 ± 55
T cell proliferation index	1.72 ± 0.11	1.87 ± 0.12
T cell immune score	11.6 ± 0.3	11.7 ± 0.3
CD8^+^CD28^+^ T cells (number/mm^3^)	181 ± 19	200 ± 20

**Table 3:** Immunological parameters showing an age-related decline

**Immunological parameters**	**Male (N = 162)**				**Female (N = 194)**				**Gender difference**
	**Slope**	**Intercept**	**R**	**p-value**	**Slope**	**Intercept**	**R**	**p-value**	**p-value**
(a) T cells^※^	−33.00	3196	0.187	0.018	−26.57	2628	0.089	0.212	0.049
(b) CD8 + T cells^※^	−15.94	1315	0.286	<0.001	−13.24	1075	0.305	<0.001	NS
(c) CD8 + CD28+ T cells^※^	−11.21	863	0.543	<0.001	−8.664	696	0.477	<0.001	0.04
(d) Naïve T cells^※^	−15.39	1205	0.089	0.261	−10.88	916	0.055	0.447	0.001
(e) T cells proliferative activity	−0.019	2.295	0.315	<0.001	−0.016	2.115	0.224	0.002	NS
(f) T cell proliferation index	−0.056	4.904	0.290	<0.001	−0.043	3.910	0.190	0.008	0.010
(g) T cell immune score	−0.137	19.3	0.261	0.001	−0.106	17.3	0.333	<0.001	0.006
(h) B cells^※^	−11.04	747	0.167	0.034	−4.307	328	0.105	0.152	0.001

※: Number / mm^3^. R: Correlation coefficient. NS: not significant.

**Table 4:** Immunological parameters showing an age-related increase

**Immunological parameters**	**Male (N = 162)**				**Female (N = 194)**				**Gender difference**
	**Slope**	**Intercept**	**R**	**p-value**	**Slope**	**Intercept**	**R**	**p-value**	**p-value**
(a) CD4 + T cells^※^	23.28	−313	<0.001	0.990	16.92	77	0.114	0.107	0.004
(b) CD4/CD8 ratio	0.087	−2.260	0.316	<0.001	0.064	−0.713	0.452	<0.001	0.001
(c) Memory T cells^※^	12.35	−141	0.114	0.153	10.11	−91	0.253	<0.001	<0.001
(d) CD4 + CD25 + T cells ^※^	1.830	−47	0.077	0.379	1.602	−33	0.100	0.233	NS
(e) NK cells^※^	17.41	−449	0.105	0.194	11.94	−186	0.268	<0.001	0.001

※: Number/mm3. R: Correlation coefficient. NS: not significant.

**Table 5:** Cytokine production and age in male and female subjects: regression analysis

**Immunological parameters**	**Men (N = 64)**				**Women (N = 49)**				**Gender difference**
	**Slope**	**Intercept**	**R**	**p-value**	**Slope**	**Intercept**	**R**	**p-value**	**p-value**
(a) IFNγ	−45.2	4698	0.721	0.071	−48.1	4677	0.122	0.397	NS
(b) IL-2	−19.2	1129	0.138	0.319	−17.2	928	0.243	0.108	NS
(c) IL-6	−293.0	18732	0.247	0.048	−488.0	29289	0.077	0.605	0.010
(d) IL-10	−51.6	3407	0.176	0.166	−28.1	1898	0.063	0.666	0.004

R: Correlation coefficient. NS: not significant.

## Discussion

The age-related decline of the immune response has a major impact on the health status of the elderly [7] and is a determinant of longevity [8]. It is well known that aging is associated with a decline in the normal function of the immune system, leading to increased susceptibility to various diseases and shortened longevity; however, specific dysfunctions in the immune system directly responsible for this have yet to be identified. Among the important factors, T cells are central to the immune response, and their function is significantly altered with increasing age. The present study confirms and extends an earlier study by Utsuyama et al. [1].

Previously, Strindhall et al. [9] reported that a reduced number of CD8^+^CD28^-^ T cells and a higher CD4^+^ T cell/CD8^+^ T cell ratio are associated with populations that survive until the age of 100 years. Moreover, Xie and McElhaney [10] reported that susceptibility to influenza infection in older adults is associated with an increase in the number of CD8^+^CD28^-^ T cells. In this study, we found that the number of CD8^+^CD28^+^ cells decreased with age, and this was associated with a decrease in the T cell proliferative response (MTS-OD_490_) and TCPI. Furthermore, the rate of decline was significantly less in women than in men.

Although CD4^+^CD25^+^ regulatory T cells are crucial to tolerance in normal individuals, the factors regulating this cell population and its function are largely unknown. In the present study, we demonstrated that the number of CD4^+^CD25^high^ regulatory T cells (TREGs) showed an increasing trend with age. Further studies are required because it is still not clear whether the age-related increase in the number of TREGs is responsible for immune dysfunction in the elderly [11].

Olsson et al. (2000) reported that a decrease in the CD4^+^ T cell/CD8^+^ T cell ratio was an important indicator of the immune risk phenotype. In the present survey, a contrasting feature was observed between CD4^+^T cells and CD8^+^T cells. The results presented here show that the number of CD4^+^ T cells was maintained at a relatively steady level or had an increasing trend with age, whereas the number of CD8^+^ T cells significantly decreased with age; therefore, the CD4^+^ T cell/CD8^+^ T cell ratio showed a distinct age-related increase. A higher percentage of CD8^+^ T cells and a decreased CD4^+^ T cell/CD8^+^ T cell ratio were observed in the Saudi male population than that in the Caucasian population [12]. Hence, it is possible that race is a factor influencing these parameters.

T cell proliferative activity is a value obtained by a determined number of T cells *in vitro*, and it does not directly reflect a value at the individual level. In order to convert the T cell proliferative activity to the individual level, we developed a new parameter, TCPI, which is calculated by the T cell proliferative activity and the number of T cells/mm^3^. Both T cell proliferative activity and TCPI showed age-related decreases in men and women. However, statistically significant differences between men and women were observed in TCPI, not in T cell proliferative activity.

In addition, we developed another novel immunological parameter, T cell immune score, which was calculated by combining 5 T cell-related parameters: number of T cells, CD4/CD8 T cell ratio, number of naïve T cells, naïve to memory T cell ratio, and TCPI. Each parameter was given a score from 3 to 1 according to the database: 3 (high level), 2 (moderate level), and 1 (low level). The summation of 5 scores was employed as the T cell immune score. It is interesting to note that the T cell immune score significantly decreased with age in both men and women, and the decline was significantly greater in men than in women.

The absolute number of total B lymphocytes increases about 3-fold from the baseline in the first year of life and progressively decreases until adulthood [13]. The present study showed that the number of B cells decreased gradually throughout life from 20 to 90 years of age, and this decline was significantly greater in men than in women.

NK cells are a key component of innate immunity and are involved in the regulation of the immune response by producing many cytokines that can activate other cellular components of innate and adaptive immunity. Although many studies have investigated the effects of age on NK cells, their conclusions remain controversial [14]. In the present study, an age-related increase was observed in the number of NK cells, and this increase was significantly higher in men than in women. It is interesting to note that Lee et al. [15] reported a higher percentage of NK cells in the Asian populations than that in the Caucasian population.

Although aging is accompanied by a progressive increase in proinflammatory cytokines, little is known about the development of age-dependent modifications in circulating cytokines. The serum levels of cytokines are usually too low to detect. Thus, in the present study, we measured the concentration of cytokines secreted into the culture supernatant by T cells in the presence of anti-CD3 monoclonal antibody. Many reports [16-18] employed the stimulation of lymphocytes by PMA and ionomycin to monitor cytokine production. Because stimulation by the anti-CD3 monoclonal antibody is more routinely used than PMA and ionomycin, we employed the former method. It is known that a shift from Type 1 to Type 2 cytokine production occurs with age; however, we could not detect such a shift. Garner and Murasko [19] reviewed over 60 studies and reported that the age-associated changes in cytokine production are inconsistent. We observed that both Th-1 type cytokines (IL-2 and IFNγ) and Th-2 type cytokines (IL-6) showed an age-related declining trend. A statistically significant decline was observed only in IL-6 in men. The rate of age-related decline in IL-10 was lesser in women than in men. Because IL-10 is known to act as an immune suppressor, a relatively lesser level of IL-10 in women could be consistent with the fact that the age-related decline of various immunological parameters is less pronounced in women than in men.

Sex hormones influence both humoral and cell-mediated immune responses, and oestrogen is one of the potential factors in this immunological dimorphism [20]. These environments are established by the cytokines that are released by immune cells, particularly T helper (Th) lymphocytes. In this regard, Pietschmann et al. [16] reported that the pattern of cytokine production is gender specific. It is possible that oestrogen deficiency after menopause induces a disturbance of peripheral tolerance through CD8^+^ T cells bearing the suppressor phenotype. In contrast, it was shown that mitochondrial oxidative stress is higher in men than in women and that the higher levels of oestrogens in women protects them against aging by upregulating the expression of antioxidants and longevity-related genes [21]. It was also demonstrated that oestrogens upregulate the expression of antioxidant enzymes via the oestrogen receptor and MAPK activation, which in turn activate the NF-kB signalling pathway, resulting in the upregulation of longevity-related gene expression [21]. The role of gender in the regulation of longevity may be linked to gender specific genetic differences, including the expression of sex hormone patterns and the changes in these patterns during an individual’s lifetime.

In humans, cytomegalovirus (CMV) infection has an enormous impact on biomarkers associated with aging of the immune system [22]. In Japan, an extensive survey of blood serum by enzyme immunoassays revealed that the percentage of CMV antibody positivity is approximately 87.2% in the whole population, showing an age-related increase. However, gender difference was not observed with regard to CMV antibody positivity and titre in each age group [23].

The essential function of T cells is the recognition of antigens and the following proliferative response. The present study confirmed that a statistically significant age-related decline was observed in T cell proliferative activity and TCPI. TCPI was the value of individual levels calculated from T cell proliferative activity, and it showed a statistically significant gender difference that was greater decline in men than in women. The lesser rate of decline in TCPI indicates that the immunological functions were relatively well preserved in elderly women than in elderly men; this finding may be consistent with the fact that women live longer than do men. As a reference, the mean lifespan of the Japanese population reported by the Japanese Ministry of Health, Labour, and Welfare is 85.5 years in women and 79.0 years in men [24].

This approximately 6 year difference in the mean lifespan may raise the question: Is this difference due to the different biological ages of the men and women? To answer the question, we compared an immunological parameter, T cell immune score, between groups of men 6 years younger than women. In other words, by removing men over 70 years and women younger than 33 years, the mean age of men was 48.2 years and that of women was 54.7 years. Then, we analysed the parameter by comparing groups of men 6 years younger than women. A significant difference was observed when comparing the original male and female groups (p < 0.012); the p-value was 0.157 when comparing groups between men 6 years younger than women. This result obtained by rearranging the groups suggested that the different life expectancy between men and women could be due to differences in biological age, and immunological parameters could be used as a marker of the biological age.

## Methods

### Blood specimens

Two millilitres of blood was collected in a tube containing ethylenediaminetetraacetic acid (EDTA-2K) for haematological analysis performed using a PENTRA80 analyser (Horiba, Kyoto, Japan). Eight millilitres of blood was collected in a cell preparation tube (vacutainer, 362761; Becton Dickinson [BD], NJ) for collecting mononuclear cells for immunological analyses.

### Subjects

A critical issue in our understanding of human aging and gender differences related to the immune system relies on the health status of a wide population range. Healthy male and female volunteers were critically selected on the basis of clinical records and laboratory examinations. A substantial number of individuals with various health problems were excluded. None of the blood donors suffered from neoplastic or autoimmune diseases; furthermore, none were receiving any medications that could influence the immune function. Routine laboratory examinations of the serum of these individuals were performed to examine the liver and kidney functions. A total of 162 men and 194 women, ranging in age from 20 to 90 years, were examined. Table 6 shows the number of male and female subjects and their ages.

**Table 6:** Number of male and female subjects

**Age (years)**	**20 - 29**	**30 - 39**	**40 - 49**	**50 - 59**	**60 - 69**	**70 – 79**	**80**	**Total**
Men	13	23	35	37	29	22	3	162
Female	44	32	36	34	18	26	4	194

### Ethical approval

This study was conducted in compliance with the Declaration of Helsinki and applicable national laws and regulations, and it was approved as no. 320 by the Ethics Committee of Tokyo Medical and Dental University. Written informed consent was obtained from all subjects.

### Flow cytometry

Mononuclear cells were stained with a combination of 2 or 3 monoclonal antibodies (mAbs) conjugated with 2 or 3 chromophores. A fluorescence-activated cell sorting flow cytometer (FACScan BD) was employed.

### Monoclonal antibodies (mABs)

The following mABs were used: fluorescein isothiocyanate (FITC) conjugated anti-CD4, FITC-conjugated anti-CD20, and FITC-conjugated anti-CD16; phycoerythrin (RD1) conjugated anti-CD3, RD1-conjugated anti-CD8, and RD1-conjugated anti-CD25; phycoerythrin-Texas Red (ECD)-conjugated anti-CD45RA and ECD-conjugated anti-CD3; phycoerythrin-cyanin 5.1 (PC5)-conjugated anti-CD28; and phycoerythrin (PE)-conjugated anti-CD56. Those mAbs were purchased from Beckman Coulter (Miami, FL). The following combinations of mAbs were used: CD3-RD1/CD20-FITC, CD4-FITC/CD8-RD1/CD45RA-ECD, CD4-FITC/CD8-RD1/CD28-PC5, CD56-PE/CD16-FITC, and CD3-ECD/CD4-FITC/CD25-RD1. These mAbs enabled us to identify T cells (CD3^+^) and various subpopulations of T cells, including CD4^+^T cells, CD8^+^T cells, naïve T cells (CD4^+^CD45RA^+^ T cells), memory T cells (CD4^+^CD45RO^+^ T cells), CD8^+^CD28^+^ T cells, B cells, and NK cells (CD56^+^CD16^+^).

### Proliferative response of T cells

The proliferative response of T cells to anti-CD3 mAb (Orthoclone OKT3; Ortho Biotec, NJ) was assessed according to the MTS method (Cell Titer 96 Aqueous One Solution Cell Proliferation Assay; Promega Co., WI). Assays were performed in microplates (3860-096, Asahi Glass Co., Japan). Cells (1 × 10^5^) were grown in 0.2 ml of RPMI 1640 medium, supplemented with 5% foetal bovine serum (FBS), and were stimulated with immobilised anti-CD3 mAb (Orthoclone OKT3; Ortho Biotec). The plates were then placed in a 5% CO_2_ incubator for 72 hrs. After incubation for 68 h, 40 μl of MTS solution (Cell Titer 96 Aqueous One Solution Cell Proliferation Assay; Promega Co.) was added into each well and the absorbance at 490 nm was recorded with a spectrophotometric plate reader; this value was used for determining the relative magnitude of T cell proliferation.

### T cells proliferation index (TCPI)

TCPI was calculated by the following equation:

$$\begin{array}{l} \text{TCPI}=T\;\text{cell}\;\text{proliferative}\;\text{activity}\\ \;\times \left(T\;\text{cell}\;\text{number}\;{\text{per mm}}^{3}/1000\right) \end{array}$$

T cell proliferative activity was based on *in vitro* data using an adjusted number of lymphocytes and does not directly reflect the activity at the whole body level. To convert the T cell proliferative activity to the whole body level, we devised a new parameter, the T cell proliferation index (TCPI) [25]. In this equation, T cell proliferative activity was obtained from the optical density (OD_490_) ranging between 0.95 and 2.0 by the above-mentioned MTS method.

### T cell immune score

Five immune parameters related to T cells were selected: number of T cells, ratio of CD4^+^ T cells to CD8^+^ T cells (CD4/CD8 ratio), number of CD4^+^CD45RA^+^ (naïve) T cells, ratio of CD4^+^CD45RA^+^ (naïve) T cells to CD4^+^CD45RO^+^ (memory) T cells (N/M ratio), and TCPI. The values of immune parameters were standardised by assigning scores of 3 (high level), 2 (moderate level), and 1 (low level) according to the database obtained from 300 healthy people. Low, moderate, and high levels were assigned to values with a cumulative frequency of less than 10%, 10% to 40%, and more than 40%, respectively. After standardization, the scores from different types of immune parameters were summed, and the numerical value obtained for each individual was termed the T cell immune score, ranging from 5 to 15.

### Cytokine production

The cells (1 × 10^6^) were grown in 0.5 ml of RPMI 1640 medium, supplemented with 10% FBS, and were stimulated with immobilised anti-CD3 mAb (Orthoclone OKT3; Ortho Biotec). The plates were then placed in a CO_2_ incubator, and the supernatant was collected 48 h later. A flow cytomix kit (BMS810FF; Bender MedSystems, Austria) was employed for the evaluation of cytokines (Interleukin (IL)-2, IL-6, IL-10, and interferon (IFN) γ), and the assessment was performed using a FACScan analyser.

### Statistical analysis

The difference in immune parameters or score between men and women was analysed by the Standardized Major Axis Test and Routines [26]. In the tables, slope, intercept, R, and the p value of the regression line are shown, and the p-values of the comparison between men and women are also shown.

## Abbreviations

SMA: Standard major axis; TCPI: T cell proliferation index; PMA: Phorbol 12-Myristate 13-acetate; MAPK: Mitogen-activated protein kinase.; CMV: Cytomegalovirus.

## Competing interests

The authors declare that they have no competing interests.

## Authors’ contributions

KH made the experimental approach and wrote the manuscript. MA performed assessment of immunological parameters. YH and MK helped writing of the manuscript. TK and TF gave important advices for the experimental approach. All authors read and approved the final manuscript.

## References

[B1] Utsuyama M Hirokawa K Kurashima C Fukayama M Inamatsu T Suzuki K Hashimoto W Sato K Differential age-change of CD4 + CD45RA + and CD4 + CD29+ T cells subsets in human peripheral blood Mech Ageing Dev 1992 63 57 68 10.1016/0047-6374(92)90016-7 1376382

[B2] Linton PJ Dorshkind K Age-related changes in lymphocytes development and function Nature Immunol 2004 5 133 139 14749784 10.1038/ni1033

[B3] Hirokawa K Utsuyama M Makinodan T Pathy MSJ, Sinclair AJ, Morley JE Immunity and ageing Principles and Practice of Geriatric Medicine 2006 4 John Wiley & Sons, Ltd 19 36

[B4] Suzuki K Hirokawa K Hatakeyama S Age-related change of distribution of immunoglobulins in containing cells in human bone marrow. Changes in patients with benign monoclonal gammopathy and multiple myeloma Virchows Arch A 1984 404 243 251 10.1007/BF00694890 6437062

[B5] Gameiro C Romao F Changes in the immune system during menopause and aging Front Biosci 2010 2 1299 1303 10.2741/e190 20515802

[B6] Barrett EL Richardson DS Sex differences in telomeres and lifespan Aging Cell 2011 10 913 921 10.1111/j.1474-9726.2011.00741.x 21902801

[B7] Pawelec G Effros RB Caruso C Remarque E Barnett Y Solana R T cells and Ageing Frontiers Biosci 1999 4 216 269 10.2741/Pawelec 10051456

[B8] Wikby A Maxson P Olsson J Johansson B Ferguson FG Changes in CD8 and CD4 lymphocyte subsets, T cell proliferation responses and non-survival in the very old: the Swedish longitudinal OCTO-immune study Mech Ageing Dev 1998 102 187 198 10.1016/S0047-6374(97)00151-6 9720651

[B9] Strindhall J Nilsson BO Löfgren S Ernerudh J Pawelec G Johansson B Wikby A No immune risk profiles among individuals who reach 100 years of age: findings form Swedish NONA immune longitudinal study Exp Gerontol 2007 42 753 761 10.1016/j.exger.2007.05.001 17606347

[B10] Xie D McElhaney JE Lower GrB + CD62Lhigh CD8 TCM effector lymphocyte response to influenza virus in older adults is associated with increased CD28null CD8+ T lymphocytes Mech Ageing Dev 2007 128 392 400 10.1016/j.mad.2007.05.001 17570460 PMC2169430

[B11] Dejaco C Duftner C Schirmer M Are regulatory T cells linked with aging? Exp.Gerontol 2006 41 339 345 10.1016/j.exger.2006.01.008 16516426

[B12] Shahabuddin S Quantitative differences in CD8+ lymphocytes, CD4/CD8 ratio, NK cells, and HLA-DR(+)-activated T cells of racially different male populations Clin Immunol Immunopathol 1995 75 168 170 10.1006/clin.1995.1067 7704975

[B13] Veneri D Franchini M Vella A Tridente G Semenzato G Pizzolo G Ortolani R Changes of human B and B-1a peripheral blood lymphocytes with age Hematol 2007 12 337 341 10.1080/10245330701255270 17654062

[B14] Solana R Tarazona R Gayoso I Lesur O Dupuis G Fulop T Innate immunosenescence: Effect of aging on cells and receptors of the innate immune system in humans Semin Immunol 2012 24 331 341 10.1016/j.smim.2012.04.008 22560929

[B15] Lee B Yap HK Chew FT Quah TC Prabhakaran K Chan GS Wong SC Seah CC Age- and sex-related changes in lymphocyte subpopulations of healthy Asian subjects: from birth to adulthood Cytometry 1996 26 8 15 10.1002/(SICI)1097-0320(19960315)26:1<8::AID-CYTO2>3.0.CO;2-E 8809475

[B16] Pietschmann P Gollob E Brosch S Hahn P Kudlacek S Willheim M Woloszczuk W Peterlik M Tragl KH The effect of age and gender cytokine production by human peripheral blood mononuclear cells and markers of bone metabolism Exp Gerontol 2003 38 1119 1127 10.1016/S0531-5565(03)00189-X 14580865

[B17] McNerian SE Rea IM Alexander HD A whole blood method for measurement of intracellular TNFa, IFNg and IL-2 expression in stimulated CD3+ lymphocytes: difference between young and elderly subjects Exp Gerontol 2002 37 227 234 10.1016/S0531-5565(01)00188-7 11772508

[B18] Alberti S Cevenini E Ostan R Capri M Salvioli S Bucci L Ginaldi L De Martinis M Franceschi C Monti D Age-dependent modification of type 1 and type 2 cytokines within virgin and memory CD4+ T cells in humans Mech Ageing Dev 2006 127 560 566 10.1016/j.mad.2006.01.014 16516272

[B19] Gardner EM Murasko DM Age-related changes in Type 1 and Type 2 cytokine in humans Biogerontol 2002 3 271 290 10.1023/A:1020151401826 12237564

[B20] Lahita RG Sex steroids and the rheumatic diseases Arthritis Rheum 1985 28 121 126 10.1002/art.1780280202 3871614

[B21] Vina J Gambini J Lopez-Grueso R Abdelaziz KM Jove M Borras C Females live longer than males: role of oxidative stress Curr Pharm Des 2011 17 3959 3965 10.2174/138161211798764942 22188448

[B22] Pawelec G Larbi A Derhovanessian E Senescence of the human immune system J Comp Pathol 2010 142 Suppl 1 S39 S44 19897208 10.1016/j.jcpa.2009.09.005

[B23] Mori T Yoshimoto R Yasue S A study of age-specific rate and titre of CMV antibody in human serum by enzyme immunoassay Immunohaematology 1986 8 243 247

[B24] Ministry of Health, Labour and Welfarehttp://www.mhlw.go.jp/english/index.html 2008

[B25] Hirokawa K Utsuyama M Ishikawa T Kikuchi Y Kitagawa M Fujii Y Nariuchi H Uetake H Sugihara K Decline of T cell-related immune functions in cancer patients and an attempt to restore them through infusion of activated autologous T cells Mech Ageing Dev 2009 130 86 91 10.1016/j.mad.2008.05.001 18555517

[B26] Falster DS Warton DI Wright IJ User’s guide to (S)MATR: Standardised Major Axis Tests & Routines 2003 http://www.bio.mq.edu.au/ecology/SMATR/23691378

